# Neuromodulation for Pelvic and Urogenital Pain

**DOI:** 10.3390/brainsci8100180

**Published:** 2018-09-29

**Authors:** Holly Roy, Ifeoma Offiah, Anu Dua

**Affiliations:** 1 Neurosurgery Department, University Hospitals Plymouth, Plymouth PL6 8DH, UK; holly.roy@nhs.net; 2 Department of Obstetrics and Gynaecology, University Hospitals Plymouth, Plymouth PL6 8DH, UK; ifyoffiah@nhs.net

**Keywords:** pelvic pain, bladder-pain syndrome, neuromodulation, posterior tibial-nerve stimulation, sacral-nerve stimulation, dorsal-root-ganglion stimulation

## Abstract

Chronic pain affecting the pelvic and urogenital area is a major clinical problem with heterogeneous etiology, affecting both male and female patients and severely compromising quality of life. In cases where pharmacotherapy is ineffective, neuromodulation is proving to be a potential avenue to enhance analgesic outcomes. However, clinicians who frequently see patients with pelvic pain are not traditionally trained in a range of neuromodulation techniques. The aim of this overview is to describe major types of pelvic and urogenital pain syndromes and the neuromodulation approaches that have been trialed, including peripheral nerve stimulation, dorsal root ganglion stimulation, spinal cord stimulation, and brain stimulation techniques. Our conclusion is that neuromodulation, particularly of the peripheral nerves, may provide benefits for patients with pelvic pain. However, larger prospective randomized studies with carefully selected patient groups are required to establish efficacy and determine which patients are likely to achieve the best outcomes.

## 1. Introduction

### 1.1. Pelvic Anatomy and Pelvic and Urogenital Pain Syndromes

Pain derived from the pelvic and urogenital region is an important clinical problem affecting both men and women. Patients may present to a range of different clinical specialties including gynecology, urology, and general surgery. Pelvic and urogenital pain syndromes include chronic pelvic pain/chronic prostatitis (CPP/CP), bladder-pain syndrome (BPS), groin/inguinal pain, and genital pain. We begin by revising relevant pelvic and urogenital anatomy before introducing the pathophysiology of individual pain syndromes.

**Figure 1:**
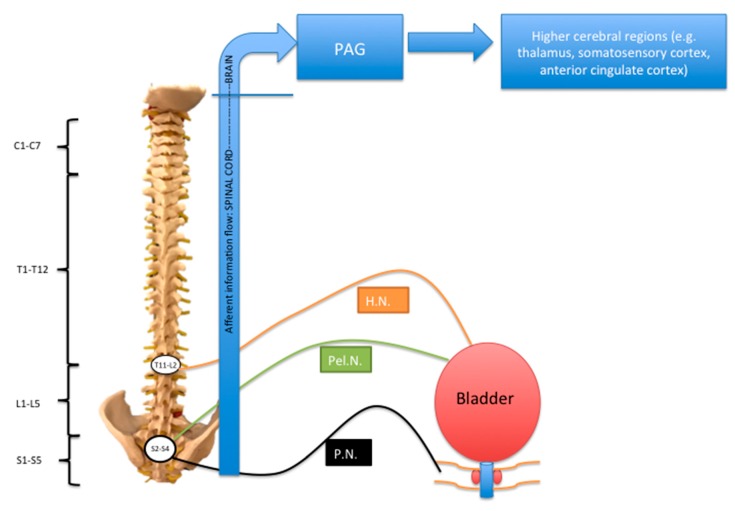
P.N.: pudendal nerve; Pel.N.: pelvic nerve; H.N. hypogastric nerve; PAG: periaqueductal grey area. Schematic to summarize afferent innervation of the lower urinary tract. The sensory fibers traveling in the pelvic and pudendal nerves have their cell bodies in dorsal root ganglia (DRGs) at the S2–S4 level. Parasympathetic fibers travel in the pelvic nerve and sympathetic fibers travel in the hypogastric nerve. Modified from Reference [1].

The true pelvis is the anatomical area between the floor of the pelvic cavity (composed of the pelvic and urogenital diaphragms) and the pelvic brim. Organs occupying the pelvis include the urinary bladder and the uterus in their empty states, the rectum, vagina, and distal parts of the male reproductive system. Both visceral and somatic nerves innervate structures within the pelvis; the innervation is complex, and in this section we will briefly summarize the main pathways for afferent (sensory) information transmission [1] (see Figure 1). Sensory afferent information from the colon, bladder, and urethra is transmitted via the splanchnic, pudendal, and pelvic nerves, whose cell bodies exist in dorsal root ganglia (DRG) at the level of the lumbosacral and thoracolumbar cord [2]. Somatic sensation to the clitoris/penis, perineal skin, and distal aspect of the anal canal are provided by branches of the pudendal nerve [3,4,5]. Furthermore, the ilioinguinal, genitofemoral, and iliohypogastric nerves provide overlapping innervation of the skin in the groin/pubic region [5]. 

Pain from the bladder is transmitted by visceral afferent nerves travelling with the sympathetic nerves via the hypogastric nerve, and also, in the case of the lower segment of the bladder, sacral parasympathetics in the pelvic nerve. Pain from the upper pelvic viscera accompanies sympathetics. Pain from the lower viscera, such as the cervix and upper vagina, travels with the parasympathetic fibers. 

Visceral afferents synapse onto second-order neurons in the dorsal horn of the spinal cord. There may be convergence of either visceral or somatic inputs onto the second-order neuron, which may potentially give rise to referred pain or cross-sensitization [6]. From the second-order neuron, information then passes either along the spinothalamic tract or the dorsal column medial lemniscus pathways to supraspinal regions responsible for processing the affective and sensory components of pain, including the periaqueductal grey area, thalamus, somatosensory cortex, and anterior cingulate cortex [7,8].

Chronic pelvic pain localized to the bladder, genitals, groin, or anorectum may be a direct result of nerve injury, inflammation, or entrapment, or may have a secondary neural component that contributes to pain amplification or maintenance. Important nerves to consider include the pudendal, thoracolumbar, ilioinguinal, iliohypogastric, genitofemoral, or obturator nerve [9]. Imaging techniques, including magnetic resonance neurography, are becoming increasingly valuable in diagnosing these conditions [10].

Afferent nociceptive plasticity and long-term plasticity in the dorsal horn of the spinal cord and supraspinal regions are important events underlying the development of chronic pain [2,8], in which the experience of pain persists after initial tissue damage has healed, and the pain has additional components such as spontaneous pain, hyperalgesia, and allodynia [7]. Many factors are involved in the development of chronic pain, including peripheral afferent sensitization, and long-term synaptic and molecular changes within the dorsal horn and brain [2,7,8,11,12,13]. Interaction with the immune system, including the microglial response, is also considered to be important in the transition to a chronic pain state [7]. Further characterization of the molecular, cellular, and network changes involved in the development of the chronic pain state are key in determining future approaches to treatment and the role of neuromodulation [7,14,15]. 

### 1.2. Major Pelvic and Urogenital Pain Syndromes (Should Include Epidemiology, Pathophysiology)

Chronic pelvic pain/chronic prostatitis (CPP/CP) is a complex pain syndrome of heterogeneous etiology. There are many inconsistencies with the way in which CPP/CP has been reported and defined, and for that reason, it is not easy to quote an accurate figure for its true incidence and prevalence [16]. However, presence of pain for at least six months is generally considered to be necessary for a diagnosis to be considered, and as a rough estimate, CPP affects 38/1000 women annually in the United Kingdom (UK), or has a prevalence of 1 million women in the UK [16], and 9.2 million women in the United States [17,18]. CP affects men, and involves symptoms of pelvic pain and/or bothersome symptoms when urinating. The U.K. prevalence of chronic prostatitis has been estimated as 8.2% [16]. A recent article supported by the International Continence Society (ICS) described nine domains to be used for the description of chronic pelvic pain, including four pelvic organ domains ((1) lower urinary tract; (2) female genital; (3) male genital; and (4) gastrointestinal); two domains representing pain perceived in the pelvis but not necessarily arising from the pelvic organs ((5) musculoskeletal; (6) neurological); and three domains that may influence pain perception ((7) psychological; (8) sexual; (9) comorbidities) [9]. In this article, we concentrate primarily on the first three pelvic-organ domains (lower urinary tract, female genital, and male genital), and the role of neurological factors in the development of pain in these domains. However, the contribution of musculoskeletal, psychological, sexual and other disease factors should not be ignored by the physician caring for patients with pelvic pain.

Chronic pain experienced in the lower-urinary-tract domain refers primarily to bladder pain syndrome (BPS)/interstitial cystitis (IC). In this article, we will refer to it as BPS. This has been defined previously by the ICS as pain or discomfort related to the urinary bladder, which is associated with other urinary symptoms, such as frequency and urgency, with the exclusion of any other diseases of the lower urinary tract [19]. Prevalence reports vary depending on the country of origin and diagnostic criteria, but are in the range of 3–4 per 100,000 in Japan to 450 per 100,000 in the Finnish population [20,21]. The precise trigger resulting in the development of BPS is still unknown. However, it is possible that bladder injury by irritant chemicals, radiation, blunt trauma, childbirth, or subclinical infection may trigger the release of inflammatory mediators and consequent disruption of the protective mucosal barrier [22,23]. Resident and recruited immune cells as well as toxic urinary solutes permeate the barrier and lead to depolarization of sensory afferents, causing bladder pain. 

Chronic pelvic pain in the male or female genital domains may be localized to the vagina, vulva, or perineum, or may involve intra-abdominal organs, including ovaries, uterus, and fallopian tubes (females), and can involve the prostate, scrotum, epididymis, testicles, or penis (males). Endometriosis, adenomyosis, adhesions, pelvic inflammatory disease, pelvic masses, peripheral pelvic neuropathies, and Tarlov cysts are potential causes [5,24,25]. Pelvic pain arising specifically from entrapment or neuropathy of the pudendal nerve is known as pudendal neuralgia (PN), which results in chronic perineal pain. Pain can extend from the perianal region to the vicinity of the scrotum/clitoris anteriorly [3]. The diagnostic criteria for pudendal neuralgia as described by the Nantes criteria include: (i) pain in the distribution of the pudendal nerve; (ii) pain experienced most significantly when sitting; (iii) pain that does not wake the patient at night; (iv) pain that is not associated with an objective sensory impairment and; (v) pain relieved by diagnostic pudendal nerve block [26]. Genital pain can also develop following lower abdominal or pelvic surgery, as in the case of scrotal pain following vasectomy [27], or, rarely, clitoral pain following midurethral sling placement [28], which is likely to be related to intraoperative nerve trauma. Pelvic pain can localize to the groin area, which may develop as a complication of inguinal hernia repair [29,30] where direct nerve damage, neuroma, postsurgical fibrosis, or compression can occur [7], resulting in pain radiating into the groin, thigh, or genitals. It is thought that up to 12% of patients may experience pain that impairs daily activity after hernia repair [31]. 

Chronic pelvic pain in the gastrointestinal domain also includes pain in the anorectal area. This can be a result of structural problems such as abscesses, anal fissures, cryptitis, and hemorrhoids [9,32], or conditions of other etiologies, such as chronic proctalgia, which may be related to pelvic floor muscle hypertonicity. Associated symptoms include diarrhea, constipation, abdominal cramps, and rectal pain. There may be associated bladder and urethral symptoms due to cross-sensitization between the bladder and colon [2]. Recognizing the possible link between colonic inflammation and bladder pain, and vice versa, is important when approaching the problem of pelvic pain.

### 1.3. Pharmacological and Non-Neuromodulatory Surgical Interventions for Pelvic and Urogenital Pain Syndromes

Initial approaches for the management of pelvic and urogenital pain syndromes are conservative and include physical therapy where indicated, simple analgesia such as paracetamol and nonsteroidal anti-inflammatories, neuropathic analgesics (particularly in patients whose pain appears to be neuropathic in nature, with a “burning” or “stabbing” quality and/or in the distribution of a known peripheral nerve) including tricyclic antidepressants, pregabalin, gabapentin, selective serotonin reuptake inhibitors, and N-methyl-D-aspartate receptor antagonists, or opioid analgesia [33,34]. Other pharmacological agents utilized for the treatment of chronic pelvic pain in women include hormonal agents (goserelin, medroxyprogesterone acetate), venoconstrictors such as ergotamine, and venomimetics such as daflon [34], while in men with chronic prostatitis, antibiotics, 5-alpha-reductase inhibitors (if benign prostatic hyperplasia is present), and phytotherapy may be used [33,35]. Psychological therapy may also be offered if indicated. Subsequent approaches in patients who are resistant to initial management and trial of pharmacotherapy tends to vary according to the pain syndrome. For bladder pain syndrome, subsequent therapy may include replacing the urothelial barrier using intravesical installation of glycosaminoglycans (such as pentosan polysulfate sodium or hyaluronic acid) [36], or use of botulinum toxin. Nerve blocks may be used if the pain is thought to be neurogenic in origin [3,5]. 

Despite a range of conservative and pharmacological options for the management of chronic pelvic and urogenital pain, (see Reference [34] for a review of specific management options for individual pelvic-pain conditions) there remains a group of patients who are resistant to pharmacological interventions. It is usually this patient group that is considered for neuromodulation, particularly if they have shown short-term responsiveness to nerve blocks. The use of neuromodulation for various chronic pelvic pain syndromes is still in its experimental phase and a matter of considerable debate [16]. Neuromodulation ranges from peripheral nerve stimulation, usually using percutaneous electrodes to target a peripheral nerve, to dorsal root ganglion stimulation, spinal cord stimulation, and brain stimulation (see Table 1). At present there is controversy surrounding the use of neuromodulation for pelvic pain syndromes. Since the majority of practitioners who see CPP in clinical practice do not have primary training in neuromodulation, the range of techniques and approaches appears overwhelming, and it may be difficult to decide when neuromodulation is appropriate and which technique to choose. The aim of this overview is to summarize the evidence for the use of different neuromodulatory approaches in the management of chronic pelvic, bladder and prostatic, groin, and genital pain syndromes. 

## 2. Neuromodulation for Pelvic-Pain Syndromes

### 2.1. Peripheral Nerve Neuromodulation

#### 2.1.1. Sacral Neuromodulation

Sacral neuromodulation (SNM) is a neuromodulatory technique whereby the sacral nerve is stimulated by an electric current via an implanted insulated lead wire placed along the sacral nerve roots, usually at the level of the S3 root. Though the precise mechanism of action of neuromodulation in relieving bladder and pelvic pain is not well understood, many publications suggest an effect on the modulation of spinal cord reflexes and brain networks, thus affecting bladder function [69,70]. First, patients are subject to a neuromodulation trial. Those who experience reduced pain with stimulation (classified as “responders”; often defined as at least 50% reduction in pain) are then allowed to progress on to implantation of a permanent implantable pulse generator (IPG), which is sited in a subcutaneous pocket in the lower quadrant of the abdomen or upper buttock and provides electrical stimulation [71]. The trial period is important as it helps prevent placement of an expensive permanent device, with its associated side effects, into a patient who may subsequently not respond to the therapy. 

SNM was first approved for use for overactive-bladder syndrome and nonobstructive urinary retention. The initial use of SNM for pelvic pain came about following reports that pain symptoms improved in patients receiving SNM for a primary complaint of urinary symptoms, such as frequency and urgency [17]. It has since been trialed off-label as a treatment for pain in chronic pelvic pain, including some cohorts with patients with a variety of pelvic pain complaints, and other studies recruiting a narrower symptom range, such as bladder pain syndrome alone. In a prospective multicenter study, Martelluci et al. (2011) [51] reported results of SNM in 27 patients with multietiological medication-resistant pelvic pain. Trial stimulation was carried out initially, with an implantation rate of 59%, and significant improvements in visual analogue scale (VAS) pain score in those patients who were implanted, both at six months and subsequent follow up (mean follow-up duration was 37 months; mean preoperative VAS was 8.1, and mean VAS at six-month follow-up was 2.1). The study team attempted to evaluate the differences between patients who had a successful trial of SNM and those who did not. They noted that all the patients who had reported some benefit from gabapentin or pregabalin (*n* = 9) went on to have definitive SNM implantation, and that all patients who reported pain following a hysterectomy had permanent implantation (*n* = 4). Furthermore, all patients who correlated pain onset with previous surgery with stapler did not experience benefit during the stimulation trial and did not go ahead with SNM implantation (*n* = 5). 

Sokal et al. (2015) [52] and Seigel et al. (2001) [17] both describe small single-center case series with good initial pain relief. The study by Seigel et al. (2001) is a prospective nonrandomized study, recruiting patients with intractable pelvic and/or urogenital pain. Results were reported from 10 patients (nine female and one male; median age 48 years; median pain duration 3 years) who all experienced >40% improvement in pain symptoms with test stimulation on an outpatient basis, and subsequently had the system implanted. Although no statistical analysis was reported, in 9 out of the 10 patients, the worst pain decreased (average decrease from 4.7 to 2.2 at long-term follow-up), and there were also improvements in other measures, such as the number of hours of worst pain and the rate of pain. However, among the 10 patients there were 27 complications reported, including local wound complications (*n* = 6), change in the location of the pain (*n* = 4), IPG site pain (*n* = 4), and implant infection (*n* = 1). Sokal et al (2015) [52] report outcomes of a prospective single-center study that recruited nine female patients with chronic pelvic pain (four as a result of failed back-surgery syndrome, and five as a result of idiopathic chronic regional pain syndrome). There was a statistically significant reduction in pain VAS at the six-month follow-up (median VAS 3 from preoperative level of 9), but reduction in efficacy at 12 months (median VAS 6), and higher than expected rate of complications, including infection and lead migration. In a mixed multicenter cohort of patients with urinary symptoms and/or perineal pain, Everaert et al. (2000) [53] also found good initial response rates to SNM (85%), which declined somewhat at a longer-term follow-up (70%). They also found that there was a significant relationship between psychiatric comorbidity and reported outcome, highlighting this as an important variable to further study in the context of SNM for chronic pelvic pain. 

SNM has also been used with good effect in two patients with intractable pelvic pain following cauda equina syndrome, and had beneficial effects on the urinary symptoms experienced by these patients (Kim et al. (2010)) [54]. In bladder-pain syndrome specifically, we reviewed three prospective studies, including a total of 137 participants, which evaluated the efficacy of sacral neuromodulation in the management of BPS. Since its introduction for the management of bladder pain, SNM has been shown to have both subjective and objective improvements in symptomatology in patients with BPS with good long-term results [55,56,57]. Results include an increase in mean voided volume, reduced pain perception, reduced urinary frequency and nocturia, and an improvement in quality of life. 

Overall, these initial trials of SNM for chronic pelvic pain suggest that it is effective for selected patients, including the BPS population, although current data relate predominantly to female rather than male patients and randomized controlled trials are difficult to identify; most studies are prospective observational trials involving patients with medication-refractory pelvic pain. Interestingly, the reported side-effect profile is relatively high, at about 3%, the most common of which being infection, lead migration, or malfunction of the pulse generator [36,72]. Other disadvantages include the fact that the procedure is expensive, which limits its use in routine clinical practice [69]. In addition, placement of the device requires specific surgical skills, which necessitates referral to a specialist and the associated waiting list. Patients also require life-long follow-ups if deemed suitable for management with sacral neuromodulation.

However, even taking disadvantages into account, the benefits afforded to medication-refractory patients by SNM strongly imply that this procedure should always be considered prior to major surgical intervention, such as augmentation procedures, urinary diversion, or cystectomy, for the purposes of pain control. Though the revision rate is high, with 49% of implanted devices requiring revision over an average follow-up of 38 months, this procedure is completely reversible, with minimal side effects of revision [73].

#### 2.1.2. Posterior Tibial Nerve Stimulation

Posterior tibial nerve stimulation (PTNS) was first described by McGuire et al. (1983) for the treatment of detrusor instability, although the original series included five patients with interstitial cystitis, of whom four improved with stimulation [74]. Early studies investigated its efficacy as a treatment for lower urinary tract symptoms [75,76,77]. However, it was observed that patients reported a concomitant improvement in levels of pain resulting in trials of PTNS as a treatment for pelvic pain. PTNS is performed in the outpatient setting on a weekly basis for a period of 12 weeks, and delivers electrical stimulation to the sacral micturition center via the sacral nerve plexus S2–4. The mechanism of action is thought to involve inhibition or modulation of the C-fiber and Aδ-afferent responses from the bladder. It is a minimally invasive procedure, which involves the placement of a fine needle into the posterior tibial nerve, approximately 5 cm cephalad to the medial malleolus [78].

The major randomized controlled trial of PTNS for chronic pelvic pain was described by Kabay et al. (2009) [37]. Of 89 patients recruited to the trial, 45 were randomised to PTNS and 44 to a sham-treatment group. Patients were randomized from a multietiological pelvic-pain group (including subjects with pain in the bladder, groin, genitals, lower abodomen, perineum, and/or perianal area), and all were male. The mean age was 37.9 years in the treatment group and 38.5 years in the sham-stimulation group, and the mean disease duration was 4.5 years (treatment group) and 3.8 years (sham-stimulation group). Stimulation was carried out in 200 μs pulses, with an amplitude range of 1–10 mA. Significant improvements in VAS for pain, urgency, and National Institutes of Health Chronic Prostatitis Symptom Index (NIH-CPSI) scores were achieved in the PTNS group at follow-up (follow-up assessments were completed at 12 weeks, immediately after the treatment) but not the sham-controlled group. The mean VAS for pain improved from 7.6 ± 0.8 to 4.3 ± 0.6 in the PTNS group, with no change in the control group (scores were 7.4 ± 0.9 before sham treatment and 7.2 ± 0.4 after sham treatment). Overall, 40% of patients in the PTNS arm of the study achieved >50% improvement in VAS. This study demonstrates moderate and statistically significant benefits of PTNS over sham stimulation in the selected population, although outcomes at longer-term follow-up are not reported. Other studies, albeit smaller in recruitment numbers, have also reported moderately positive outcomes for PTNS in chronic pelvic pain [38,39,40], using similar stimulation parameters to those described by Kabay et al. [37]. Two other randomized controlled trials, both including only female patients with mixed etiology chronic pelvic pain, reported significant improvements in VAS for the PTNS group, but not the control group at a 12-week follow-up [39,40], although the statistical significance was not maintained at the six-month follow-up [39]. However, marked improvements in pain have not been reported by all studies; for example, in a prospective cohort study, enrolling male and female patients with mixed etiology pelvic pain, and a mean age of 51 years, only 21% of patients reported an improvement in VAS of >50% and, although the improvement in VAS for the group was statistically significant, the magnitude of change in VAS was small (from 6.5 at baseline to 5.4 after treatment) [41]. The authors note that improvement was better in patients with certain pain distributions (e.g., perineal, perianal, and vaginal) and that increasing the frequency of stimulation from once per week to more regularly might result in better outcomes based on findings from the overactive-bladder literature [76]. 

PTNS has also been tested as an experimental treatment for BPS. However, there are conflicting reports on its efficacy. A study by Congregado and colleagues reported significant improvements in all irritative lower urinary tract symptoms and hypogastric pain after 10 weeks of treatments with PTNS [42]. The study was a prospective observational follow-up study in 51 female patients with lower-urinary-tract irritative symptoms who had experienced no prior response to anticholinergic medications. All patients appeared to report hypogastric pain prior to treatment, but only 33% reported hypogastric pain at follow-up (mean follow-up duration was 21 months). Unfortunately, it is not clear in the paper how hypogastric pain was evaluated [76], and it appears that patients were recruited on the basis of their irritative symptoms and not primarily bladder pain. In contrast with these positive findings, Zhao et al. (2008) reported no significant change in the VAS pain score of BPS patients after a 10-week trial of PTNS in an open prospective trial, though significant improvement in bladder volume was noted, along with complete pain resolution in a single subject and statistically significant improvements in other secondary measures, such as the Interstitial Cystitis Problem Index and the O’Leary/Sant Interstitial Cystitis Problem Index [44]. Subsequently, this result was echoed in a trial by Regab and colleagues where they reported no effect on BPS symptoms following intermittent PTNS after 0, six, and 12 weeks of treatment [45]. Using a slightly different treatment approach, Baykal et al. found that PTNS when used in combination with glycosaminoglycan replacement therapy (intravesical heparin) was effective in improving pain scores and bladder capacity in refractory BPS patients (10 female, two male) who had failed “more than one classical therapy” [43]. There was no control arm in this study to compare the effects of PTNS + intravesical heparin with intravesical heparin alone; however, positive results suggest that this should be investigated further.

Overall, the main advantage of PTNS is that it is minimally invasive, with only mild side effects (predominantly pain at the insertion site and mild bleeding or bruising [38,40,44]) compared with other types of neuromodulation, and, as a result of that, patients tend to find it acceptable [44]. There appears to be a moderate benefit of PTNS for pelvic pain in medication-refractory patients [38,39,40,42,43], but the benefits may tail off over time, since the stimulator is not permanently implanted, unlike SNM. Reduction in efficacy at long-term follow-up was found by Istek et al. [34], and it is clear that more long-term follow-up studies are needed to investigate this further. Large prospective randomized controlled trials that are able to compare the effect of PTNS with sham stimulation and also identify phenotypes within the pelvic-pain spectrum that respond more favorably to stimulation would be an important next step. Further trials of combination therapy (for example, glycosaminoglycan replacement + PTNS) may also be of benefit in more fully exploring the role of PTNS in pelvic pain. 

#### 2.1.3. Pudendal Nerve Stimulation

Pudendal nerve stimulation can be successful for pelvic pain when the pain is identified as being perineal in nature, and if the pain is associated with features of pudendal neuralgia. As with sacral neuromodulation, the technique can be carried out as a two-stage procedure, with a lead positioned at the pudendal nerve for test stimulation, and connected to an implantable pulse generator if the test stimulation proves successful. Peters et al. (2015) [79] conducted a retrospective review in which 19 patients who had undergone pudendal neuromodulation at a single center for pudendal neuralgia were sent questionnaires to evaluate outcome. All patients had had some improvement in pain at the time of implantation. Only 10 out of 19 patients returned the questionnaires; of these, seven reported some improvement (four reported slight improvement, one reported moderate improvement, and two reported marked improvement). However, pain medications received more favorable assessments, with six out of 10 patients describing a marked improvement. In a case series of three patients, Carmel et al. (2009) [26] reported more favorable outcomes, with one patient pain-free at two-year follow-up, and two patients reporting 80% pain relief. However, numbers are small and further studies are needed to strengthen the evidence for this treatment strategy. In cohorts of patients with BPS, pudendal neuromodulation has been shown in several studies and case reports to be effective in alleviating pain, especially in patients who have failed management with sacral neuromodulation. However, this method is new, has limited evidence, and is therefore not routinely practiced. We reviewed three studies with a total number of 102 subjects that evaluated the role of pudendal nerve stimulation in the management of patients with BPS. The first, a retrospective study on 84 patients concluded that pudendal neuromodulation could be recommended in patients who are refractory to sacral neuromodulation: 93% of patients who had previously failed sacral neuromodulation responded to pudendal stimulation [80]. When compared to sacral nerve stimulation in a blinded randomized trial design study, this approach was reported to lead to significantly greater reduction in bladder pain and irritative urinary symptoms in complex BPS patients [80,81]. Finally, pudendal neuromodulation was described in a case report, in combination with sacral neuromodulation, to produce excellent results for the treatment of complex pelvic neuropathy [82]. The pudendal nerve may thus play a more important role in the management of BPS than is currently recognized in daily clinical practice. 

#### 2.1.4. Stimulation of Other Peripheral Nerves

Although PTNS and sacral neuromodulation are by far the most common nerve stimulation techniques for chronic pelvic pain, followed by pudendal neuromodulation, neuromodulation of other peripheral nerves, including the genitofemoral, ilioinguinal, iliohypogastric, and vagus nerves, has been successfully performed in small numbers of patients for intractable inguinal pain [46,47,48]. Carayannopoulos et al. (2009) [46] published outcomes for two patients; the first had medication-refractory pain in the inguinal, genital, and thigh regions, which had temporarily responded to ilioinguinal nerve blocks and pulsed radiofrequency ablation of the ilioinguinal nerve, and the second had groin pain that was not completely relieved by medications. Patients reported 90% and 85%–95% pain alleviation seven days after implantation; however, longer-term follow-up data were not provided. A study by Shaw et al. (2016) [47] included six patients with chronic neuropathic inguinal and genital pain (four male, two female, mean pain duration 4.6 years). All patient had undergone trials of other therapies, including medication and nerve blocks, for the treatment of their pain. Five out of six patients had sustained benefit with stimulation at long-term follow-up (average follow-up duration was 22 months) and two patients had a VAS pain score of zero at that point. Testicular pain following hydrocele surgery has also been reported as responding well to stimulation of the cutaneous branch of the ilioinguinal, and the genital branch of the genitofemoral nerves in a case report by Rosendal et al. 2012 [49]. Pain intensity reduced from 9/10 to 2/10 at seven-month follow-up in this patient. 

Stimulation of the vagus nerve has been attempted for the control of pelvic pain, based on evidence that the vagus nerve plays a role in visceral nociception. In a study of 15 female subjects, Napadow et al. 2012 [50] investigated the effect of respiratory-gated auricular vagal afferent nerve stimulation on pain relief in patients with chronic pelvic pain. The study used a randomized crossover design comparing a single session of respiratory-gated auricular vagal afferent nerve stimulation with a single session of auricular stimulation, which was nonvagal. They found that patients undergoing the respiratory-gated auricular vagal afferent nerve stimulation had significantly less anxiety than the nonvagal stimulation group, and that there was a trend towards reductions in evoked pain intensity and temporal summation of evoked pain in the respiratory-gated auricular vagal afferent nerve stimulation group. 

### 2.2. Dorsal-Root-Ganglion Stimulation

The DRG is a collection of cell bodies of sensory neurons that is located bilaterally at each spinal level encased within the bony vertebral structure. As part of the anatomical pathway involved in pain transmission, electrical stimulation of the DRG has been explored as a treatment for chronic pain [59].

DRG stimulation, with the stimulating electrodes at L1 and L2 level, has been reported in a single case of intractable, medication-resistant pelvic girdle pain, with a 43% reduction in pain at six-month follow-up [60]. DRG stimulation has also been described for groin pain. Sensory input to DRGs at T11-L3 corresponds to the groin area. In a multicenter study of DRG stimulation for chronic pain, 10/10 patients with postherniorrhaphy pain had a successful stimulation trial, and the mean reduction in VAS score at follow-up was 76.8 ± 8.2% [58]. Larger prospective studies are awaited.

### 2.3. Spinal-Cord Stimulation

Spinal-cord stimulation (SCS) a common neurostimulation approach for the treatment of chronic pain, first reported early in the second half of the twentieth century [83], which involves surgical laminotomy and placement of electrodes in the epidural space between T9 and T11 for lower-limb pain. Its mechanism of action is thought to involve modulation of pain transmission in the spinal-cord dorsal horn, in addition to manipulating autonomic function and interacting with supraspinal pain-processing mechanisms. Although there is good evidence for its use in severe pain associated with failed back-surgery syndrome, chronic regional pain syndrome, and neuropathic pain, far less is known about its efficacy for visceral pain and pelvic pain. However, there are a small number of studies describing its use in this context [61,62,63]. Buffenoir et al. (2015) [61] report outcomes following SCS at the conus medullaris in a prospective dual-center study enrolling a total of 27 patients with pudendal neuralgia, recruited over a 13-month period. Twenty out of 27 patients were classified as ‘responders’ (>50% reduction of maximum pain or >50% increase of sitting time before pain onset). The estimated percentage improvement at long-term follow-up was 55% with a mean tripling of sitting time. Short-term complications included one infection and one suboptimal electrode fixation but no long-term complications were described. This technique has been recently replicated in a small case series with good results [63]. SCS at T7–9 levels for groin/inguinal pain has also been reported as beneficial in small groups of patients with postherniorrhaphy groin pain, [64,65]. However, SCS-induced parasthesias may not always fully cover the groin area, and in this case, a combination of SCS with peripheral field stimulation may be useful [66]. Despite these studies, the standard of evidence for SCS in the context of pelvic pain remains of a fairly low quality and further research is needed to define the appropriate context for this technique.

### 2.4. Brain Stimulation for Pelvic-Pain Syndromes: Existing Evidence and Future Directions

The peripheral afferent drive is known to be important in chronic pain, including chronic pelvic-pain syndromes. As a demonstration of this, intravesical installation of alkalinized lidocaine has been shown to have benefits for selected patients with bladder-pain syndrome [84], and is thought to have its effects by silencing the afferent pain drive [6]. However, in patients who do not effectively respond to such treatment, it is reasonable to assume that central mechanisms contribute substantially to their experience of pain. In such cases of chronic pelvic pain where central sensitization plays a key role in the development and maintenance of the chronic pain state, peripheral approaches to neuromodulation may fail to address the root cause of the problem, and a strategy of central neuromodulation may be more effective for symptom control. Indeed, a test of the contribution of the peripheral afferent drive, such as intravesical lidocaine, might become a tool for selecting candidates for central, supraspinal neuromodulation. Brain stimulation techniques for the control of pain include motor cortex stimulation (MCS), in which epidural electrodes are sited over the motor cortex, and deep brain stimulation (DBS) in which electrodes are implanted at targets within the brain itself, including the periaqueductal/periventricular grey area, the ventral posterolateral and ventral posteromedial thalamus, and the anterior cingulate cortex. In the case of chronic pelvic pain, MCS stimulation for pelvic and perineal pain has been described at case-report level to provide improvement in medication-refractory cases that have failed an alternative neuromodulation trial, or those for which peripheral or spinal neuromodulation is contraindicated [67]. Far more trial data is needed to determine if this should be considered routinely in refractory pelvic pain. Similarly, there may be a potential role for DBS in the control of pelvic pain. At the time of writing, there did not appear to be any studies describing individual outcomes following DBS for pelvic, groin, or genital pain, although DBS has been performed for these indications, with outcomes reported as averages within larger series, (e.g., Reference [68]), but in which it is not possible to identify specific outcomes for pelvic-pain patients.

## 3. Conclusions

### 3.1. What We Know 

Chronic pelvic pain is a major area of unmet clinical need, with massive associated morbidity and health costs, and encompassing a wide range of different pain syndromes. The underlying pathophysiology is heterogeneous and likely to involve both peripheral and central mechanisms. Neuromodulation is an emerging option for patients with refractory pelvic pain, and both PTNS and SNM are recognized as potential treatments. Our conclusion is that peripheral neuromodulation, such as PTNS, SNM, or pudendal nerve stimulation, should be considered in patients whose pain is refractory to medication, particularly if they have shown some response to a nerve block. PTNS has a better side-effect profile than SNM, but its effects seem to be more short-lived. Other neuromodulation techniques, such as DRG stimulation and spinal cord stimulation, are still highly experimental but may also be considered in selected patients. 

### 3.2. What We Do Not Know

There are many gaps in the current literature regarding neuromodulation for urogenital and chronic pelvic pain. Firstly, knowledge about underlying pathophysiological mechanisms of pain in chronic pelvic pain syndromes, particularly the role of peripheral and central mechanisms maintaining the pain state, is still incomplete. There is a shortage of large, randomized controlled trials of neuromodulation therapies for chronic pelvic pain, and it is therefore difficult to fully assess efficacy. Furthermore, most studies focus on female patients, and lack long-term follow-up, so the long-term effectiveness and relevance for male patients is not known. There are no direct comparisons between neuromodulation types, and little is understood about which subgroups and phenotypes might respond better to different types of neuromodulation. Finally, knowledge about the potential of spinal cord and brain stimulation for pelvic and urogenital pain is limited to case-report level only and further studies are needed.

### 3.3. Limitations of This Overview

This overview is limited in that it is not a formal systematic review. We have also only included work published in the English language, which may have limited the article’s scope. Finally, we did not attempt to contact the authors of studies that included pelvic pain patients within larger series without specifically providing a breakdown of results for pelvic pain patients. Therefore, knowledge available from these studies, for example, the work on DBS for pelvic pain, was not accessed here. 

## Figures and Tables

**Table 1:** Summary of types of neuromodulation technique and application for pelvic pain.

Neuromodulation Technique	Description	Indications	Advantages	Disadvantages	References
Percutaneous posterior tibial nerve stimulation	Placement of a fine needle into the posterior tibial nerve approximately 5 cm cephalad to the medial malleolus	Bladder pain syndrome (BPS), Chronic pelvic pain/Chronic prostatitis (CPP/CP)	Minimally invasive, low-risk, easier to perform, relatively cost-effective, no long-term follow-up needed	Need for patients to attend clinic weekly for 12 weeks to complete treatment. Minor side effects including mild pain and bleeding.	[37,38,39,40,41,42,43,44,45]
Implantable peripheral nerve stimulation devices	Implantation of insulated wire connected to implantable pulse generator to stimulate selected nerve (e.g., pudendal nerve)	Pudendal nerve (BPS, CPP/CP, pudendal neuralgia) genitofemoral, ilioinguinal, iliohypogastric (groin/genital pain)	Good specificity of effect	Requires technical skill, risk of infection, lead migration, and need for long-term follow-up	[46,47,48,49,50]
Sacral neuromodulation	Stimulation of sacral nerve roots by an electric current via an implanted insulated lead wire placed usually along the S3 sacral nerve root	CPP/CP, BPS, groin pain	Relatively widely used, so good evidence base to guide treatment.	Infection, lead migration or malfunction of the pulse generator or pain at the pulse generator site. Challenges in electrode placement.	[17,51,52,53,54,55,56,57]
Dorsal root ganglion stimulation	Implantation of an electrode connected to implantable pulse generator over the dorsal root ganglion	Pelvic girdle pain, groin pain	Long-term analgesic effects and specific anatomical targeting of the pain relief, as well as fewer changes in analgesic effect with changes in body posture	Requires technical skill, risk of infection, lead migration, and need for long-term follow up. Fewer large well-conducted trials into DRG stimulation for pelvic pain due to the fact that it is relatively new as a technique for this indication	[58,59,60]
Spinal cord stimulation	Implantation of an electrode over the dorsal spinal cord in the epidural space	CPP/CP, particularly pudendal neuralgia	Good efficacy in limited number of reported cases	Small number of studies carried out.	[61,62,63,64,65,66]
Motor cortex stimulation	Stimulation of motor cortex by placement of electrode in epidural space	CPP	May be an option in patients for whom peripheral or spinal neuromodulation was unsuccessful or contraindicated	Limited evidence	[67]
Deep brain stimulation	Stimulation of specific intracranial target by stereotactically placed electrodes	N/A	May be an option in patients for whom peripheral or spinal neuromodulation was unsuccessful or contraindicated	Limited evidence	[68]

## References

[1] Kanai A., Andersson K.E. (2010). Bladder Afferent Signaling: Recent Findings. J. Urol..

[2] Grundy L., Brierley S.M. (2018). Cross-organ sensitization between the colon and bladder: To pee or not to pee?. Am. J. Physiol. Gastrointest. Liver Physiol..

[3] Panicker J.N., Manji H., Clare J.F., Jalesh N.P., Emmanuel A. (2010). Neuromuscular Disorders. Pelvic Organ Dysfunction in Neurological Disease: Clinical Management and Rehabilitation.

[4] Graziottin A., Gambini D. (2015). Anatomy and physiology of genital organs–women. Handb. Clin. Neurol..

[5] Elkins N., Hunt J., Scott K.M. (2017). Neurogenic pelvic pain. Phys. Med. Rehabil. Clin. N. Am..

[6] Gebhart G.F., Bielefeldt K. (2016). Physiology of visceral pain. Compr. Physiol..

[7] Inoue K., Tsuda M. (2018). Microglia in neuropathic pain: Cellular and molecular mechanisms and therapeutic potential. Nat. Rev. Neurosci..

[8] Bliss T.V.P., Collingridge G.L., Kaang B.K., Zhuo M. (2016). Synaptic plasticity in the anterior cingulate cortex in acute and chronic pain. Nat. Rev. Neurosci..

[9] Rana N., Drake M.J., Rinko R., Dawson M., Whitmore K. (2018). The fundamentals of chronic pelvic pain assessment, based on international continence society recommendations. Neurourol. Urodyn..

[10] Weissman E., Boothe E., Wadhwa V., Scott K., Chhabra A. (2017). Magnetic resonance neurography of the pelvic nerves. Semin. Ultrasound CT MR.

[11] Chen A., De E., Argoff C. (2018). Small fiber polyneuropathy is prevalent in patients experiencing complex chronic pelvic pain. Pain Med..

[12] Eller-Smith O.C., Nicol A.L., Christianson J.A. (2018). potential mechanisms underlying centralized pain and emerging therapeutic interventions. Front. Cell. Neurosci..

[13] Levesque A., Riant T., Ploteau S., Rigaud J., Labat J.J. (2018). Clinical criteria of central sensitization in chronic pelvic and perineal pain (convergences PP criteria): Elaboration of a clinical evaluation tool based on formal expert consensus. Pain Med..

[14] Tam J., Loeb C., Grajower D., Kim J., Weissbart S. (2018). Neuromodulation for chronic pelvic pain. Curr. Urol. Rep..

[15] Tutolo M., Ammirati E., Heesakkers J., Kessler T.M., Peters K.M., Rashid T., Sievert K.D., Spinelli M., Novara G., Van der Aa F. (2018). Efficacy and safety of sacral and percutaneous tibial neuromodulation in non-neurogenic lower urinary tract dysfunction and chronic pelvic pain: A systematic review of the literature. Eur. Urol..

[16] Baranowski A.P., Lee J., Price C., Hughes J. (2014). Pelvic pain: A pathway for care developed for both men and women by the British Pain Society. Br. J. Anaesth..

[17] Siegel S., Paszkiewicz E., Kirkpatrick C., Hinkel B., Oleson K. (2001). Sacral nerve stimulation in patients with chronic intractable pelvic pain. J. Urol..

[18] Parasar P., Ozcan P., Terry K.L. (2017). Endometriosis: Epidemiology, diagnosis and clinical management. Curr. Obstetrics Gynecol. Rep..

[19] Abrams P., Cardozo L., Fall M., Griffiths D., Rosier P., Ulmsten U., van Kerrebroeck P., Victor A., Wein A. (2002). The standardisation of terminology of lower urinary tract function: Report from the Standardisation Sub-committee of the International Continence Society. Neurourol. Urodyn..

[20] Leppilahti M., Tammela T.L., Huhtala H., Auvinen A. (2002). Prevalence of symptoms related to interstitial cystitis in women: A population-based study in Finland. J. Urol..

[21] Rosenberg M.T., Hazzard M. (2005). Prevalence of interstitial cystitis symptoms in women: A population-based study in the primary care office. J. Urol..

[22] Graham E., Chai T.C. (2006). Dysfunction of bladder urothelium and bladder urothelial cells in interstitial cystitis. Curr. Urol. Rep..

[23] Keay S.K., Birder L.A., Chai T.C. (2014). Evidence for bladder urothelial pathophysiology in functional bladder disorders. BioMed Res. Int..

[24] Cheong Y., Saran M., Hounslow J.W., Reading I.C. (2018). Are pelvic adhesions associated with pain, physical, emotional and functional characteristics of women presenting with chronic pelvic pain? A cluster analysis. BMC Womens Health.

[25] Hunter C.W., Stovall B., Chen G., Carlson J., Levy R. (2018). Anatomy, pathophysiology and interventional therapies for chronic pelvic pain: A review. Pain Physician.

[26] Carmel M., Lebel M., Tu le M. (2010). Pudendal nerve neuromodulation with neurophysiology guidance: A potential treatment option for refractory chronic pelvi-perineal pain. Int. Urogynecol. J..

[27] Manikandan R., Srirangam S.J., Pearson E., Collins G.N. (2004). Early and late morbidity after vasectomy: A comparison of chronic scrotal pain at 1 and 10 years. BJU Int..

[28] Christofferson M., Barnard J., Montoya T.I. (2015). Clitoral pain following retropubic midurethral sling placement. Sex. Med..

[29] Kehlet H., Jensen T.S., Woolf C.J. (2006). Persistent postsurgical pain: risk factors and prevention. Lancet.

[30] Poobalan A.S., Bruce J., Smith W.C., King P.M., Krukowski Z.H., Chambers W.A. (2003). A review of chronic pain after inguinal herniorrhaphy. Clin. J. Pain.

[31] Aasvang E., Kehlet H. (2005). Surgical management of chronic pain after inguinal hernia repair. Br. J. Surg..

[32] Chiarioni G., Asteria C., Whitehead W.E. (2011). Chronic proctalgia and chronic pelvic pain syndromes: Etiologic insights and treatment options. World J. Gastroenterol..

[33] Fall M., Baranoski A., Elneil S., Engeler D., Hughes J., Messelink E.J., Oberpenning F., de Williams A.C. (2010). EAU guidelines on chronic pelvic pain. Eur. Urol..

[34] Cheong Y.C., Smotra G., Williams A.C.D.C. (2014). Non-surgical interventions for the management of chronic pelvic pain. Cochrane Database Syst. Rev..

[35] Nickel J.C. (2004). The three as of chronic prostatitis therapy: Antibiotics, alpha-blockers and anti-inflammatories. What is the evidence?. BJU Int..

[36] Wang J., Chen Y., Chen J., Zhang G., Wu P. (2017). Sacral Neuromodulation for refractory bladder pain syndrome/interstitial cystitis: A global systematic review and meta-analysis. Sci. Rep..

[37] Kabay S., Kabay S.C., Yucel M., Ozden H. (2009). Efficiency of posterior tibial nerve stimulation in category IIIB chronic prostatitis/chronic pelvic pain: A Sham-Controlled Comparative Study. Urol. Int..

[38] Kim S.W., Paick J.S., Ku J.H. (2007). Percutaneous posterior tibial nerve stimulation in patients with chronic pelvic pain: A preliminary study. Urol. Int..

[39] Istek A., Gungor Ugurlucan F., Yasa C., Gokyildiz S., Yalcin O. (2014). Randomized trial of long-term effects of percutaneous tibial nerve stimulation on chronic pelvic pain. Arch. Gynecol. Obstet..

[40] Gokyildiz S., Kizilkaya Beji N., Yalcin O., Istek A. (2012). Effects of percutaneous tibial nerve stimulation therapy on chronic pelvic pain. Gynecol. Obstet. Investig..

[41] Van Balken M.R., Vandoninck V., Messelink B.J., Vergunst H., Heesakkers J.P., Debruyne F.M., Bemelmans B.L. (2003). Percutaneous tibial nerve stimulation as neuromodulative treatment of chronic pelvic pain. Eur. Urol..

[42] Congregado Ruiz B., Pena Outeirino X.M., Campoy Martinez P., Leon Duenas E., Leal Lopez A. (2004). Peripheral afferent nerve stimulation for treatment of lower urinary tract irritative symptoms. Eur. Urol..

[43] Baykal K., Senkul T., Sen B., Karademir K., Adayener C., Erden D. (2005). Intravesical heparin and peripheral neuromodulation on interstitial cystitis. Urol. Int..

[44] Zhao J., Bai J., Zhou Y., Qi G., Du L. (2008). Posterior tibial nerve stimulation twice a week in patients with interstitial cystitis. Urology.

[45] Ragab M.M., Tawfik A.M., Abo El-enen M., Elnady M., El-Gamal O.M., El-Kordy M., Gameel T., Rasheed M. (2015). Evaluation of percutaneous tibial nerve stimulation for treatment of refractory painful bladder syndrome. Urology.

[46] Carayannopoulos A., Beasley R., Sites B. (2009). Facilitation of percutaneous trial lead placement with ultrasound guidance for peripheral nerve stimulation trial of ilioinguinal neuralgia: A technical note. Neuromodulation.

[47] Shaw A., Sharma M., Zibly Z., Ikeda D., Deogaonkar M. (2016). Sandwich technique, peripheral nerve stimulation, peripheral field stimulation and hybrid stimulation for inguinal region and genital pain. Br. J. Neurosurg..

[48] Al Tamimi M., Davids H.R., Barolat G., Krutsch J., Ford T. (2008). Subcutaneous peripheral nerve stimulation treatment for chronic pelvic pain. Neuromodulation.

[49] Rosendal F., Moir L., de Pennington N., Green A.L., Aziz T.Z. (2013). Successful treatment of testicular pain with peripheral nerve stimulation of the cutaneous branch of the ilioinguinal and genital branch of the genitofemoral nerves. Neuromodulation.

[50] Napadow V., Edwards R.R., Cahalan C.M., Mensing G., Greenbaum S., Valovska A., Li A., Kim J., Maeda Y., Park K. (2012). Evoked Pain Analgesia in Chronic Pelvic Pain Patients using Respiratory-Gated Auricular Vagal Afferent Nerve Stimulation. Pain Med..

[51] Martellucci J., Naldini G., Carriero A. (2012). Sacral nerve modulation in the treatment of chronic pelvic pain. Int. J. Colorectal Dis..

[52] Sokal P., Zielinski P., Harat M. (2015). Sacral roots stimulation in chronic pelvic pain. Neurol. Neurochir. Pol..

[53] Everaert K., De Ridder D., Baert L., Oosterlinck W., Wyndaele J.J. (2000). Patient satisfaction and complications following sacral nerve stimulation for urinary retention, urge incontinence and perineal pain: A multicenter evaluation. Int. Urogynecol. J. Pelvic Floor Dysfunct..

[54] Kim J.H., Hong J.C., Kim M.S., Kim S.H. (2010). Sacral nerve stimulation for treatment of intractable pain associated with cauda equina syndrome. J. Korean Neurosurg. Soc..

[55] Al-Zahrani A.A., Elzayat E.A., Gajaweski J.B. (2011). Long-term outcome and surgical intervewntions after sacral neuromodulation implant for lower urinary tract symptoms: 14-year experience at 1 centre. J. Urol..

[56] Gajewski J.B., Al-Zahrani A.A. (2011). The long-term efficacy of sacral neuromodulation in the management of intractable cases of bladder pain syndrome: 14 Years of experience in one centre. BJU Int..

[57] Ghazwani Y.Q., Elkelini M.S., Hassouna M.M. (2011). Efficacy of sacral neuromodulation in treatment of bladder pain syndrome: Long-term follow-up. Neurourol. Urodyn..

[58] Schu S., Gulve A., EIDabe S., Baranidharan G., Wolf K., Demmel W., Rasche D., Sharma M., Klase D., Jahnichen G. (2015). Spinal cord stimulation of the dorsal root ganglion for groin pain—A retrospective review. Pain Pract..

[59] Liem L., Russo M., Huygen F.J.P.M., van Buyten J.P., Smet I., Verrils P., Cousins M., Brooker C., Levy R., Deer T., Kramer J. (2013). A Multicenter, Prospective trial to assess the safety and performance of the spinal modulation dorsal root ganglion neurostimulator system in the treatment of chronic pain. Neuromodulation.

[60] Rowland D.C., Wright D., Moir L., FitzGerald J.J., Green A.L. (2016). Successful treatment of pelvic girdle pain with dorsal root ganglion stimulation. Br. J. Neurosurg..

[61] Buffenoir K., Rioult B., Hamel O., Labat J.J., Riant T., Robert R. (2015). Spinal cord stimulation of the conus medullaris for refractory pudendal neuralgia: A prospective study of 27 consecutive cases. Neurourol. Urodyn..

[62] Kapural L.N.S., Janicki T.I., Mekhail N. (2006). Spinal cord stimulation is an effective treatment for the chronic intractable visceral pelvic pain. Pain Med..

[63] Simopoulos T.Y.R., Gill J.S. (2018). Treatment of chronic refractory neuropathic pelvic pain with high frequency 10 kilohertz spinal cord stimulation. Pain Pract..

[64] Elias M. (2000). Spinal cord stimulation for post-herniorrhaphy pain. Neuromodulation.

[65] Yakovlev A.E., Resch B.E. (2009). Treatment of intractable abdominal pain patient with Bannayan-Riley-Ruvalcaba syndrome using spinal cord stimulation. WMJ.

[66] Lepski G., Vahedi P., Tatagiba M.S., Morgalla M. (2013). Combined spinal cord and peripheral nerve field stimulation for persistent post-herniorrhaphy pain. Neuromodulation.

[67] Louppe J.M., Nguyen J.P., Robert R., Buffenoir K., de Chauvigny E., Riant T., Pereon Y., Labat J.J., Nizard J. (2013). Motor cortex stimulation in refractory pelvic and perineal pain: Report of two successful cases. Neurourol. Urodyn..

[68] Boccard S.G., Pereira E.A., Moir L., Aziz T.Z., Green A.L. (2013). Long-term outcomes of deep brain stimulation for neuropathic pain. Neurosurgery.

[69] Abello A., Das A.K. (2018). Electrical neuromodulation in the managemnt of lower urinary tract dysfunction: Evidence, experience and future prospects. Ther. Adv. Urol..

[70] Kessler T.M., de Wachter S. (2017). Neuromodulation of lower urinary tract dysfunction. Urol. A.

[71] Lucas M.G., Bedretdinova D., Bosch J.L.H.R., Burkhard F., Cruz F., Nambiar A.K., Nilsson C.G., de Ridder D.J.M.K., Tubaro A., Pickard R.S. (2012). Guidelines on urinary incontinence. Eur. Assoc. Urol..

[72] Zabihi N., Mourtzinos A., Maher M.G., Raz S., Rodriguez L.V. (2008). Short-term results of bilateral S2-S4 sacral neuromodulation for the treatment of refractory interstitial cystitis, painful bladder syndrome, and chronic pelvic pain. Int. Urogynecol. J. Pelvic Floor Dysfunct..

[73] Donon L., Robert G., Ballanger P. (2014). Sacral neuromodulation: results of a monocentric study of 93 patients. Prog. Urol..

[74] McGuire E.J., Zhang S.C., Horwinski E.R., Lytton B. (1983). Treatment of motor and sensory detrusor instability by electrical stimulation. J. Urol..

[75] Stoller M.L. (1999). Afferent nerve stimulation for pelvic floor dysfunction. Eur. Urol..

[76] Klingler H.C., Pycha A., Schmidbauer J., Marberger M. (2000). Use of peripheral neuromodulation of the S3 region for treatment of detrusor overactivity: A urodynamic-based study. Urology.

[77] Govier F.E., Litwiller S., Nitti V., Kreder K.J., Rosenblatt P. (2001). Percutaneous afferent neuromodulation for the refractory overactive bladder: Result of a multicenter study. J. Urol..

[78] Gupta P., Ehlert M.J., Siris L.T., Peters K.M. (2015). Percutaneous tibial nerve stimulation and sacral neuromodualtion: An update. Curr. Urol. Rep..

[79] Peters K.M., Killinger K.A., Jaeger C., Chen C. (2015). Pilot study exploring chronic pudendal neuromodulation as a treatment option for pain associated with pudendal neuralgia. Low Urin. Tract Symptoms.

[80] Peters K.M., Killinger K.A., Boguslawski B.M., Boura J.A. (2010). Chronic pudendal neuromodulation: Expanding available treatment options for refractory urologic symptoms. Neurourol. Urodyn..

[81] Peters K.M., Feber K.M., Bennett R.C. (2007). A prospective, single-blind, randomized crossover trial of sacral vs pudendal nerve stimulation for interstitial cystitis. BJU Int..

[82] Armstrong G.L., Vancaillie T.G. (2016). Combined site-specific sacral neuromodulation and pudendal nerve release surgery in a patient with interstitial cystitis and persistent arousal. BMJ Case Rep..

[83] Shealy C.N., Mortimer J.T., Reswick J.B. (1967). Electrical inhibition of pain by stimulation of the dorsal columns: Preliminary clinical report. Anesth. Analg..

[84] Nickel J.C., Moldwin R., Lee S., Davis E.L., Henry R.A., Wyllie M.G. (2009). Intravesical alkalinized lidocaine (PSD597) offers sustained relief from symptoms of interstitial cystitis and painful bladder syndrome. BJU Int..

